# All-cause mortality in patients with treatment-resistant depression: a cohort study in the US population

**DOI:** 10.1186/s12991-019-0248-0

**Published:** 2019-09-30

**Authors:** Gang Li, Daniel Fife, Grace Wang, John J. Sheehan, Robert Bodén, Lena Brandt, Philip Brenner, Johan Reutfors, Allitia DiBernardo

**Affiliations:** 1 Real World Evidence, Statistics & Decision Sciences, Janssen Research & Development, 920 US Highway 202 S, Raritan, NJ 08869 USA; 2 Department of Epidemiology, Janssen Research & Development, 920 US Highway 202 S, Raritan, NJ 08869 USA; 3 Janssen Scientific Affairs, LLC, Titusville, NJ USA; 4 Department of Neuroscience, Psychiatry, Uppsala University, Uppsala, Sweden; 5 Centre for Pharmacoepidemiology, Department of Medicine Solna, Karolinska Institutet, Karolinska University Hospital, Stockholm, Sweden

**Keywords:** Major depressive disorder, Mortality, Treatment-resistant depression

## Abstract

**Background:**

Treatment-resistant depression (TRD) may represent a substantial proportion of major depressive disorder (MDD); however, the risk of mortality in TRD is still incompletely assessed.

**Methods:**

Data were obtained from Optum Clinformatics™ Extended, a US claims database. Date of the first antidepressant (AD) dispensing was designated as the index date for study entry and 6 months prior to that was considered the baseline period. Patients with MDD aged ≥ 18 years, index date between January 1, 2008 and September 30, 2015, no AD claims during baseline, and continuous enrollment in the database during baseline were included. Patients who started a third AD regimen after two regimens of appropriate duration were included in the TRD cohort. All-cause mortality was compared between patients with TRD and non-TRD MDD using a proportional hazards model and Kaplan–Meier estimate with TRD status being treated as a time-varying covariate. The model was adjusted for study year, age, gender, depression diagnosis, substance use disorder, psychiatric comorbidities, and Charlson comorbidity index.

**Results:**

Out of 355,942 patients with MDD, 34,176 (9.6%) met the criterion for TRD. TRD was associated with a significantly higher mortality compared with non-TRD MDD (adjusted HR: 1.29; 95% CI 1.22–1.38; *p* < 0.0001). Survival time was significantly shorter in the TRD cohort compared with the non-TRD MDD cohort (*p* < 0.0001).

**Conclusions:**

Patients with TRD had a higher all-cause mortality compared with non-TRD MDD patients.

## Background

Approximately 4.4% of the world’s population suffers from depression at any given time, making depression the largest cause of disability world-wide (7.5% of all years lived with disability [YLDs]) [1]. In the US, depression is the second largest contributor to YLDs, after back pain [2]. Among American adults, the life-time prevalence of major depressive disorder (MDD) is 20.6% [3]. Extensive research over decades has found an association between depression and increased mortality [4–9]. A recent meta-analysis found that depression was associated with a 50% increase in mortality [10].

Though depression is a known risk factor for suicide [11], suicide alone does not entirely explain the increased mortality in depression. Depression may elicit pathophysiological changes such as peripheral inflammation, oxidative stress, and cardio-metabolic conditions that contribute to the development of chronic somatic diseases that increase the risk of mortality [5]. Various effects of depression, such as decreased treatment adherence, sedentary lifestyle, smoking, unhealthy diet, and other mental comorbidities may also contribute to the increased mortality [5]. Moreover, in patients with pre-existing chronic diseases, comorbid depression may be an independent risk factor for mortality [12–16].

Treatment-resistant depression (TRD) may represent up to 60% of patients with depression, depending on the response or remission criteria [17]. Results of the sequenced treatment alternatives to relieve depression (STAR*D) trial showed a 50% cumulative remission rate in outpatients with MDD after two different antidepressant (AD) regimens of adequate dose and duration, and the likelihood of remission substantially decreased after two regimens [18, 19]. The failure of two adequate AD regimens is a common criterion for defining TRD [20, 21].

The literature has suggested that TRD may be associated with an increased risk of mortality. Based on a review of clinical studies, Carney et al. concluded that TRD is associated with a higher cardiovascular mortality as compared with treatment responders [23]. In a Swedish study, Reutfors et al. reported that patients with TRD had a 35% higher all-cause mortality than non-TRD MDD patients [24]. However, the association seems complex, as several risk factors for TRD, as well as several detrimental outcomes from TRD, may themselves be associated with increased mortality. Among these are social and functional impairment, comorbidities such as substance use disorders (SUD), anxiety disorders, and personality disorders, frequent and recurrent episodes of depression, and frequent hospitalizations [17, 18, 22].

Taken together, the available body of research suggests that patients with TRD may have a higher all-cause-mortality than other patients with MDD. This association should be studied in different populations to strengthen the body of evidence and explore the generalizability. Such investigations should ideally be conducted in large-sized cohorts with data that allows for long term follow-up, and which contains information on socio-demographic and clinical variables known to be associated both with TRD and with increased mortality. Therefore, the current study was conducted to estimate the all-cause mortality in TRD patients compared with non-TRD MDD patients using administrative claims data in the US.

## Methods

### Data source

Data for the current analysis were obtained from Optum Clinformatics™ Extended Data Mart (CEDM), a claims database that contains covered lives with combined benefit structure that includes both medical and prescription coverage. The CEDM database captures information regarding health care costs, resource utilization, quality, and effectiveness. The database includes approximately 15 million covered affiliate lives annually. The population is heavily weighted to a commercial health plan population, but also includes a Medicare Advantage population.

The CEDM also contains mortality data of patients. The National Technical Information Service (NTIS) receives Death Master File (DMF) data from the Social Security Administration (SSA) and disseminates them on behalf of SSA. Effective November 1, 2011, the source of the mortality data changed to the NTIS’s Limited Access DMF, which contains data on decedents who died fewer than 3 years ago. So, death reports were missing from the data when death occurs outside medical facilities after November 1, 2011. Therefore, these data may not be suitable for absolute mortality estimates; nevertheless, assuming the data are missing at random with respect to TRD status, they are useful for comparative assessment.

### Study design and sample selection

**Fig. 1:**
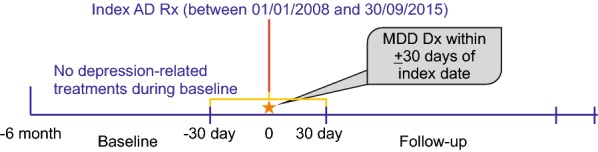
Study design. *AD* antidepressant, *MDD* major depressive disorder, *Rx* prescription, *Dx* diagnosis

Date of the first AD dispensing was considered as the index date for inclusion in the cohort. A period of 6 months prior to the index date was set as baseline (Fig. 1). International Classification of Diseases, Ninth and Tenth Revisions, Clinical Modification (ICD-9-CM and ICD-10-CM) diagnosis codes were used for data retrieval related to different diagnoses (Table 1).

**Table 1:** ICD-9 and ICD-10 codes of the conditions mentioned in this study

Condition	ICD-9	ICD-10
Major depressive disorder	296.2×, 296.3×, 300.4, 309.0, 309.1, 309.28, 311	F32–F34
Depression comorbidity		
Anxiety	300.0×, 300.2×, 300.3	F40, F41, F42
Personality disorder	301.×	F34, F60, F21
Post-traumatic stress disorder	309.81	F43
Self-harm	E950–E959	X60–X84, Y87.0, T14.91
Suicide ideation	V62.84	R45.851

Patients aged ≥ 18 years who filled a prescription for AD medication between January 1, 2008 and September 30, 2015, who were diagnosed with ICD 296.2× (major depressive disorder single episode), 296.3× (major depressive disorder recurrent episode), 300.4 (dysthymic disorder), 309.0 (adjustment disorder with depressed mood), 309.1 (prolonged depressive reaction), 309.28 (adjustment disorder with mixed anxiety and depressed mood), 311 (depressive disorder, not elsewhere classified) within ± 30 days from the index date, and had no claims for an AD with continuous enrollment in the insurance plan throughout the baseline period were included in the analysis. Among patients with an ICD depression diagnosis code 300.4, 309.0, 309.1, or 311 there was also the restriction that only those who had at least two consecutive AD dispenses with a gap of ≤ 30 days (to ensure some level of compliance) post index date were included. Patients diagnosed with psychosis, mania and bipolar disorder, or dementia during the baseline period, and those who had received treatment with lithium, thyroid hormone, antipsychotics, mood stabilizers (carbamazepine, lamotrigine, valproate), electroconvulsive therapy or repetitive transcranial magnetic stimulation during the baseline period were excluded. The classification of ICD-codes into an overall category of MDD varies between studies. We chose to follow the ICD-9 Code-Drug Match POS Prior Authorization Guideline of UnitedHealthcare (2014) because the data source of Optum is from the insurance policies sponsored by UnitedHealthcare [25].

An MDD patient who started a third AD regimen after two AD regimens of adequate duration was classified as a patient with TRD. The first medication had to be an AD but the second and the third could be an AD either with or without an augmentation therapy (see Additional file 1). Because claims databases do not capture reasons for medication change, the failure of a medication was determined operationally by quantifying an ‘adequate duration’ for that medication with a lower and an upper limit. Results of data exploration suggest that about 20% of patients had a duration of first AD < 28 days before change (switched to or augmented with the second AD) and about 15% had a duration of second AD < 28 days, and it is unlikely that a patient and caregiver would continue a regimen with inadequate results for more than 3 months. We therefore set an upper limit of 180 days on the time from the start of the first AD regimen to the start of the third AD regimen [21] (Additional file 2). The starting date for the third AD was defined as the TRD start date. Patients were followed up from the TRD start date until death, or were censored if they dropped out of the insurance plan; were diagnosed with psychosis, mania/bipolar disorder or dementia; or until the end of data collection.

Use of the Optum database was reviewed by the New England Institutional Review Board (IRB) and was determined to be exempted from IRB approval, as this project does not involve human subject research. Confidentiality of patient records was always maintained. The study report contains aggregate data only and does not identify individual patients or physicians.

### Assessment

The outcome of interest was all-cause mortality. Survival probability was estimated, and the relative risk of all-cause mortality was compared between the TRD and non-TRD MDD cohorts. Relative risk of mortality in the TRD and non-TRD MDD cohorts was further assessed on subgroups based on gender, age (categorized as 18–24, 25–34, 35–44, 45–54, 55–64, 65+ years), study year (from 2008 to 2015), depression diagnosis (categorized as diagnosis with MDD, dysthymic disorder, adjustment disorder with depressed mood, or depressive disorder), psychiatric comorbidity status (yes/no), self-harm status (yes/no), and substance use disorder (SUD) status (yes/no) at baseline.

### Statistical analysis

Patient characteristics and outcome measures for the TRD and the non-TRD MDD cohorts were summarized using descriptive statistics. All-cause mortality between TRD and non-TRD MDD patients was compared using a proportional hazards model with TRD status being treated as a time-varying covariate. The model was adjusted for the following covariates evaluated during the baseline period: study year, age, gender, depression diagnosis, SUD, other psychiatric comorbidities (diagnosis of anxiety, post-traumatic stress disorder, or personality disorder), and Charlson comorbidity index (CCI). The CCI is a method of measuring the burden of comorbidities, in which each comorbidity category is assigned a weight, based on the adjusted risk of mortality [26]. A composite score of morbidity, the Quan-Charlson comorbidity index (QCI) was used [27]. To assess the relative risk of mortality on the TRD and non-TRD MDD cohorts in a subgroup, this subgroup variable was not included in the model adjustment. Relative risk of all-cause mortality was estimated as the hazard ratio (HR) with 95% confidence interval (CI). The Kaplan–Meier survival estimates of the TRD and non-TRD MDD cohorts were compared using the log-rank test.

## Results

A total of 355,942 pharmacologically treated MDD patients were included in the analysis. At baseline, the mean age was 46.4 years, most of the patients were women (62.4%), 3.0% had SUD, 0.5% had a diagnosis of self-harm, and 37.1% had other psychiatric comorbid diagnoses. During the study, 34,176 (9.6%) patients met the criteria for TRD (Table 2).

**Table 2:** Demographic and baseline characteristics

	All MDD patients *N* = 355,942	TRD patients *N* = 34,176
Age, mean years (SD)	46.4 (18.01)	44.5 (17.35)
Charlson comorbidity score, mean (SD)	0.063 (0.372)	0.183 (0.630)
Age groups, years (%)		
18–24	40,681 (11.4)	4686 (13.7)
25–34	68,137 (19.1)	6669 (19.5)
35–44	69,687 (19.6)	6970 (20.4)
45–54	63,632 (17.9)	6344 (18.6)
55–64	46,306 (13.0)	4369 (12.8)
≥ 65	67,499 (19.0)	5138 (15.0)
Gender, *n* (%)		
Women	222,165 (62.4)	21,443 (62.7)
Depression subtype, *n* (%)		
Major depressive disorder (ICD 296.2, 296.3×)	130,379 (36.6)	14,622 (42.8)
Dysthymic disorder (ICD 300.4)	43,115 (12.1)	3836 (11.2)
Adjustment disorders with depressive symptoms (ICD 309.0, 309.1, 309.28)	11,758 (3.3)	950 (2.8)
Other depressive disorder (ICD 311)	170,690 (48.0)	14,768 (43.2)
Substance use disorder, *n* (%)		
Yes	10,582 (3.0)	1263 (3.7)
Self-harm, *n* (%)		
Yes	1610 (0.5)	275 (0.8)
Other psychiatric comorbidities, *n* (%)		
Yes	131,963 (37.1)	13,743 (40.2)

*SD* standard deviation, *MDD* major depressive disorder, *TRD* treatment-resistant depression

**Fig. 2:**
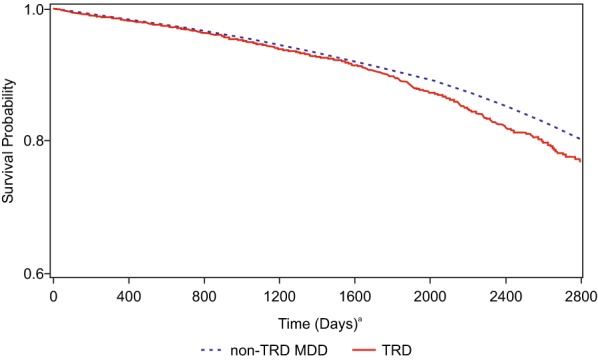
Kaplan–Meier plot. ^a^Day 0 was the first antidepressant dispensing day for non-TRD MDD and the TRD onset date for TRD patients

Survival time was significantly shorter in the TRD cohort compared with the non-TRD MDD cohort (log-rank *p* < 0.0001) (Fig. 2). Patients with TRD had a significantly higher overall all-cause mortality (HR: 1.29; 95% CI 1.22, 1.38) compared with non-TRD MDD patients. The all-cause mortality in the TRD cohort was significantly higher within most of the subgroups (Table 3). The subgroups whose point estimates for HRs stood out were: study year 2015 (HR: 2.28; 95% CI 1.57, 3.31), age group 25–34 years (HR: 2.35; 95% CI 1.69, 3.26), and self-harm (yes) (HR: 2.02; 95% CI 1.00, 4.05). For other subgroups defined by gender, depression diagnosis, psychiatric comorbidity, and SUD, the point estimates for HR did not vary much across the categories (Table 3).

**Table 3:** Hazard ratios of all-cause mortality in the TRD cohort vs. the non-TRD MDD cohort

	Event number/*N*	HR (95% CI)	*p* value
Overall	13,124/355,942	1.29 (1.22, 1.38)	< 0.0001
Year			
2008	3331/59,997	1.33 (1.19, 1.49)	< 0.0001
2009	2684/49,834	1.25 (1.09, 1.44)	0.0015
2010	2076/44,272	1.33 (1.14, 1.55)	0.0003
2011	1534/44,567	1.20 (0.99, 1.45)	0.0644
2012	1239/43,556	1.48 (1.20, 1.82)	0.0002
2013	1058/41,992	1.49 (1.18, 1.87)	0.0008
2014	775/40,490	1.47 (1.09, 1.98)	0.0105
2015	427/31,234	2.28 (1.57, 3.31)	< 0.0001
Gender			
Women	6837/222,165	1.34 (1.23, 1.46)	< 0.0001
Men	6287/133,777	1.25 (1.14, 1.37)	< 0.0001
Age group			
18–24	108/40,681	1.50 (0.84, 2.68)	0.1698
25–34	232/68,137	2.35 (1.69, 3.26)	< 0.0001
35–44	486/69,687	1.38 (1.04, 1.83)	0.0282
45–54	1032/63,632	1.31 (1.07, 1.61)	0.0097
55–64	1745/46,306	1.35 (1.15, 1.57)	0.0001
65+	9521/67,499	1.24 (1.15, 1.34)	< 0.0001
Depression subtype			
Major depressive disorder (ICD 296.2, 296.3×)	4358/130,379	1.33 (1.20, 1.47)	< 0.0001
Dysthymic disorder (ICD 300.4)	1007/43,115	1.40 (1.13, 1.74)	0.0024
Adjustment disorders (ICD 309.0, 309.1, 309.28)	420/11,758	1.31 (0.90, 1.89)	0.1586
Other depressive disorder (ICD 311)	7339/170,690	1.25 (1.15, 1.37)	< 0.0001
Other psychiatric comorbidities			
No	9880/223,979	1.29 (1.20, 1.39)	< 0.0001
Yes	3244/131,963	1.32 (1.18, 1.49)	< 0.0001
Self-harm			
No	13,067/354,332	1.29 (1.22, 1.38)	< 0.0001
Yes	57/1610	2.02 (1.00, 4.05)	0.0495
Substance use disorder			
No	12,519/345,360	1.30 (1.22, 1.38)	< 0.0001
Yes	605/10,582	1.24 (0.95, 1.61)	0.1136

Hazard ratios are presented overall and stratified on subgroups

*TRD* treatment-resistant depression, *HR* hazard ratio, *CI* confidence interval

## Discussion

The current large cohort study showed a significantly increased all-cause mortality in patients with TRD compared with non-TRD MDD patients, after adjusting for several risk factors. MDD is associated with significantly elevated risk of early death, which may be attributed to the association between MDD and a variety of chronic physical health disorders, and with increased suicide risk [28, 29]. Results of the current study suggest that the increased mortality in patients with MDD is concentrated among those with TRD.

The proportion of patients with TRD in the current analysis (9.6%) was similar to that reported in two other claims database analyses (6.6%, 11%) [30, 31], but was substantially lower than the prevalence concluded from the STAR*D trial, in which approximately 50% of the patients did not have complete remission with two AD trials [32]. The prevalence reported in the claims database studies may not be comparable to the prevalence reported in the STAR*D study due to differences in populations and in TRD criteria. In retrospective claims database studies, the TRD criteria are based only on the count of medication regimens, but the STAR*D study was also able to consider clinical assessments [33]. In addition, patients receiving protocol-defined treatment, as in STAR*D study, may have moved faster through therapies than would be typical of real-world treatment patterns. Either or both of these, or the different patient populations, may explain the higher proportion of TRD in the STAR*D study.

Although no previous study has investigated the mortality risk in patients with TRD in the US population, a recent cohort study in Swedish patients, using a similar definition of TRD as in the present study, showed that patients with TRD had a 35% higher all-cause mortality compared with non-TRD MDD patients (HR: 1.35; 95% CI 1.21, 1.50) [24]. Findings of the current study, which showed a 29% higher all-cause mortality in patients with TRD compared with non-TRD MDD patients (HR: 1.29; 95% CI 1.22, 1.38), are in agreement with the Swedish study. Reutfors et al. reported that the increase in mortality comparing TRD vs. non-TRD MDD was most prominent in the younger subgroup (aged 18–29 years) (HR: 2.03; 95% CI 1.55, 2.64); in the current study, the relative increase in mortality was highest among TRD patients between 25 and 34 years of age (HR: 2.35; 95% CI 1.69, 3.26).

Multiple factors could contribute to increased mortality among patients with TRD. Walker et al. observed increased mortality among patients with mental health disorders overall due to unnatural, natural, and unknown causes [8], and this increased mortality risk has previously been observed in TRD [34, 35]. Although patients in both cohorts had MDD, prior data have shown patients with TRD have longer episodes compared with those with non-TRD MDD [30] and risk of suicide is elevated during a depressive episode [36]. Therefore, the increased mortality could in part reflect the higher proportion of the follow-up period the TRD cohort likely spends in an episode in this analysis. Exiting an episode and attaining remission could be particularly clinically important for TRD patients because, in contrast to non-TRD MDD patients, an increased risk of suicide has been observed in TRD patients even at mild levels of depression symptoms [37]. Additionally, Amos et al., reported an increased proportion of patients with TRD have an inpatient stay compared with Non-TRD MDD patients [38]. Multiple reports have found a large increase in suicide risk following a mental-health-related inpatient stay [34, 39–41]. It is not likely that the mental-health-related inpatient stay is causally related to the increased risk of suicide, but that other acute aspects of the patient’s depression contribute to both the risk of inpatient stay and suicide. Because mortality from natural causes is also associated with TRD, suicide can only partly explain increased mortality. It is possible that TRD modifies lifestyle factors that increase the risk of developing physical comorbidities that impact mortality, or lead to poorer management of these comorbidities once they occur. It is also likely that physical comorbidities among patients with MDD increase the risk of developing TRD, but we attempted to adjust for that possibility in this analysis. In totality, our findings add to the literature suggesting the potential importance of helping patients with depression manage their symptoms and exit an episode, and TRD patients face greater hurdles in achieving these goals.

Limitations of the current study include uncertain disease coding in claims databases, which are usually captured for billing purposes, and may not reflect clinically and systematically verified definitions of medical conditions. It is not possible to determine from claims data whether regimens were changed due to lack of efficacy or other reasons such as lack of tolerability or poor adherence or any other reasons. In addition, this study did not examine the adequacy of the antidepressant doses and included patients with depression who may not have met the criteria for MDD. Considering that the data did not include patients’ entire histories, the notion of incident cases in this study was limited to the current episode. Because many individuals reside in claims database for fewer than 3 years, the portions of the curves in Fig. 2 that describe the mortality experience of patients over substantially longer time periods, may not be generalizable. Though we attempted to adjust for confounding, some confounding may have remained. For example, confounding by medical comorbidities may not have been entirely removed by adjustment for the Charlson comorbidity index. Additionally, the data source did not have information about MDD severity, cause of death, or suicide; so, effect of these variables on outcome could not be estimated. Finally, the population of Optum CEDM is primarily representative of commercial claims patients with lower proportion of Medicare population, hence the study population is likely to have higher socio-economic status compared with the overall US population.

Strengths of the current study include use of a large data set collected from an administrative database that provided medication exposure information based on dispensing details of medications, adjusting for several potential confounders, and being based on information from real-world patients who were receiving usual care.

## Conclusions

Patients with TRD may have a significantly greater risk of all-cause mortality compared with non-TRD MDD patients. The current study has added to the emerging literature as, to best of our knowledge, it is the first study in the US on increased all-cause mortality in patients with TRD as compared with non-TRD MDD patients. Results are likely to be fairly generalizable as the study was conducted using a large data set that reflects current clinical practices in the US.

## Supplementary information

**Additional file 1.** List of antidepressant medications and minimum adequate dose. Provides a detailed list of antidepressants and their minimum adequate dose considered during the analysis.

**Additional file 2.** Method of determination of adequate duration of antidepressant therapy. Detailed explanation of algorithm used to estimate adequate duration of antidepressant therapy.

## Data Availability

The data sets during and/or analyzed during the current study available from the corresponding author on reasonable request.

## Abbreviations

AD: Antidepressant
CCI: Charlson comorbidity index
CEDM: Clinformatics™ Extended Data Mart
CI: Confidence interval
DMF: Death master file
HR: Hazard ratio
ICD: International Classification of Diseases
IRB: Institutional Review Board
MDD: Major depressive disorder
NTIS: National Technical Information Service
SSA: Social Security Administration
STAR*D: Sequenced treatment alternatives to relieve depression
SUD: Substance use disorder
TRD: Treatment-resistant depression
YLD: Years lived with disability
