# Supplementary material for: All-cause mortality in patients with treatment-resistant depression: a cohort study in the US population
Source: Ann Gen Psychiatry. 2019 Sep 30;18:23. doi: 10.1186/s12991-019-0248-0 (PMC6771113; doi:10.1186/s12991-019-0248-0)
Supplement: Supplementary file 1 — Additional file 1. List of antidepressant medications and minimum adequate dose. Provides a detailed list of antidepressants and their minimum adequate dose considered during the analysis. [file 12991_2019_248_MOESM1_ESM.docx]

# List of antidepressant medications and minimum adequate dose

| **Antidepressant medication** | **Minimum daily adequate dose^1^** |
| --- | --- |
| **SSRIs** |  |
| Citalopram | 20 mg |
| Escitalopram | 10 mg |
| Fluvoxamine^2^ | 50 mg |
| Fluvoxamine, continuous release^2^ | 100 mg |
| Fluoxetine | 20 mg |
| Paroxetine | 20 mg |
| Paroxetine, extended release | 12.5 mg |
| Sertraline | 50 mg |
| Vilazodone^2^ | 10 mg |
| **DNRI** |  |
| Bupropion | 150 mg |
| **SNRIs** |  |
| Desvenlafaxine | 50 mg |
| Duloxetine | 60 mg |
| Levomilnacipran^2^ | 20 mg |
| Milnacipran^2^ | 12.5 mg |
| Venlafaxine | 37.5 mg |
| **Serotonin modulators** |  |
| Nefazodone | 50 mg |
| Trazodone | 150 mg |
| Vortioxetine^2^ | 10 mg |
| **Norepinephrine-serotonin modulator** |  |
| Mirtazapine | 15 mg |
| **Tricyclics and tetracyclics** |  |
| Amitriptyline | 25 mg |
| Amoxapine^2^ | 50 mg |
| Clomipramine^2^ | 25 mg |
| Doxepin | 25 mg |
| Desipramine | 25 mg |
| Imipramine | 25 mg |
| Maprotiline | 75 mg |
| Nortriptyline | 25 mg |
| Protriptyline | 10 mg |
| Trimipramine | 25 mg |
| **MAOIs** |  |
| Isocarboxazid | 10 mg |
| Moclobemide | 150 mg |
| Opipramol |  |
| Phenelzine | 15 mg |
| Selegiline transdermal | 6 mg |
| Tranylcypromine | 10 mg |
| **Other selected medications^3^** |  |
| Agomelatine |  |
| Mirtazapine |  |
| Mianserin |  |
| Nefazadone |  |
| Noxiptiline |  |
| Olanzapine-fluoxetine | 25 mg |
| Pipofezine |  |
| Reboxetine |  |
| Tianeptine |  |
| MAOIs = monoamine oxidase inhibitors; NDRIs = norepinephrine-dopamine reuptake inhibitors; SNRIs = serotonin-norepinephrine reuptake inhibitors; SSRIs = selective serotonin reuptake inhibitors. | |
| **Notes:** | |
| 1. Starting doses were based on the recommended starting dose indicated in the American Psychiatric Association (APA) Practice Guidelines for Treatment of Patients with Major Depressive Disorder, 3^rd^ edition, 2010 (https://psychiatryonline.org/pb/assets/raw/sitewide/practice_guidelines/guidelines/mdd.pdf). | |
| 2. Starting doses for other antidepressant medications not included in the APA Practice Guidelines for Treatment of Patients with Major Depressive Disorder were based on the starting doses indicated in the label (http://www.accessdata.fda.gov/scripts/cder/drugsatfda/index.cfm). | |
| 3. Other selected medications includes an antidepressant-antipsychotic combination treatments indicated for treatment resistant depression | |

# List of augmentation medications

**Antipsychotic medications**

| Aripiprazole |
| --- |
| Cariprazine |
| Clozapine |
| Iloperidone |
| Olanzapine |
| Paliperidone |
| Quetiapine |
| Risperidone |
| Ziprasidone |
| **Other augmentation medications** |
| Lithium |
| Thyroid hormone (T3 and T4) |
