# Supplementary material for: All-cause mortality in patients with treatment-resistant depression: a cohort study in the US population
Source: Ann Gen Psychiatry. 2019 Sep 30;18:23. doi: 10.1186/s12991-019-0248-0 (PMC6771113; doi:10.1186/s12991-019-0248-0)
Supplement: Supplementary file 2 — Additional file 2. Method of determination of adequate duration of antidepressant therapy. Detailed explanation of algorithm used to estimate adequate duration of antidepressant therapy. [file 12991_2019_248_MOESM2_ESM.docx]

**METHOD OF DETERMINATION OF ADEQUATE DURATION OF ANTIDEPRESSANT THERAPY**

The adequate duration was not straightforward, as claims databases do not have records of reasons for change of medications of patients. As a result, the failure of a medication was determined operationally by whether a medication had an ‘adequate duration’ before switch to or augmented with the next medication. The adequate duration was quantified with a lower and an upper limit.

A pilot study evaluated the distributions of duration of the first and second AD medications before change to the next AD medication. The results of study suggested that about 20% patients with a duration of first AD <28 days before change (switched to or augmented with the second AD medication) and about 15% with a duration of second AD medication < 28 days, on the other hand, about 45% patients had their first and second AD medications change more than 180 days. It was believed that the effectiveness of new AD medications should present in 28 days. As a result, the lower and upper limits were set to be 28 and 180 days, respectively.

A MDD patient was defined as TRD if the patient had received 3 antidepressant regimens, the first medication had to be an antidepressant but the second and the third could be either an antidepressant with or without an augmentation medication. The following figure describes the algorithm to determine treatment failure of A (the first line treatment) based on its duration before drug B is introduced within one MDD episode. Drug A was considered a failure if B was introduced between 29 to 180 days or if B was augmented with A starting on day 15. In other words, the lower limit of the adequate duration was 29 and an upper limit 180 days; the lower limit was lowered to 15 days if B was augmented with A. Similar algorithm applies for the failure of the second line treatment. The third AD medication start date was the TRD date. If a patient received ECT or rTMS treatment, that patient was also considered a TRD patient; the TRD date was the earliest date of the third AD medication, ECT or rTMS date.
